# Market integration and economic growth

**DOI:** 10.1371/journal.pone.0294467

**Published:** 2023-11-28

**Authors:** Shiqi Lyu, Zexian Chen, Simei Pan, Lianhua Liu

**Affiliations:** 1 School of Finance, Guangdong University of Finance and Economics, Guangzhou, Guangdong, China; 2 Desautels Faculty of Management, McGill University, Montreal, Quebec, Canada; 3 Department of Business Administration, Guangzhou Huashang College, Guangzhou, Guangdong, China; Qufu Normal University, CHINA

## Abstract

This study empirically examines the interplay between market integration and economic growth across nine cities within the Pearl River Delta urban agglomeration. The findings indicate that the city cluster’s market integration negatively impacts regional economic development and has a negligible effect on the surrounding areas. In response, the research recommends the elimination of market trade barriers and a reduction in local protectionism within the city cluster. Additionally, infrastructure enhancement is essential to leverage the distinct comparative advantages of each city within the Pearl River Delta urban agglomeration. An efficient collaboration mechanism is crucial to amplify the collective economic potency of the region.

## 1. Introduction

The Pearl River Delta(PRD) city cluster area contains nine cities: Guangzhou, Shenzhen, Foshan, Huizhou, Dongguan, Zhuhai, Zhaoqing, Jiangmen, and Zhongshan. The PRD city cluster is located in the lower reaches of the Pearl River, with outstanding location advantages. It is adjacent to Hong Kong and Macao and is separated from Southeast Asia by the sea. It has excellent geographical conditions for convenient transportation, with the vast inland economic hinterland at its back and the excellent ports of Shenzhen and Guangzhou at the mouth of the Pearl River. The PRD city cluster has a highly developed economy. In 2021, the PRD urban agglomeration created 80.876% of the total GDP of Guangdong Province with 30% of its land area, with a total GDP value of USD 1,518.022 billion.

City clusters are an important force in promoting regional economic development. As one of the most dynamic economic zones in China, the PRD city cluster is an important engine for the economic development of Guangdong and even China. The degree of market integration of urban agglomerations is the core element affecting the economic growth in the region. The PRD city cluster still has the problem of unbalanced and insufficient economic development among cities in its market economy development process. There are still problems such as an inadequate market economy system and bottlenecks in the spatial flow of goods and factors. How to promote the construction of a higher level of integration of the market and thus promote the quality of economic development has become a key concern of the academic community today. Therefore, this paper explores the impact of market integration on economic growth, based on an empirical study of the level of development of market economy integration in the PRD city cluster. This study is expected to contribute to promoting the integrated development of the PRD urban agglomerations and to improving the theory of an innovative model of economic development with coordinated regional development oriented to urban agglomerations, thus providing new ideas for the high-quality development of China’s market economy.

The purpose of this paper is to elucidate the characteristics of the spatial connection between economic development and market economy of the nine cities in the PRD city cluster and the characteristics of the spatial development evolution of economic development and market economy integration of these cities under the background of the integrated development of the market economy and to explore the influencing factors of the evolution of their spatial development. A model of the relationship between economic development and the development of market integration was constructed to analyze the level of economic development with GDP per capita as an explanatory variable. Market integration analysis in terms of commodity market integration, labor market integration, and capital market integration as explanatory variables. Variables such as the degree of Economic Openness(*Open*_*it*_), Human Capital(*Pers*_*it*_), Consumption Level(*Con*_*it*_), Scale of Government Spending(*Gov*_*it*_), and Social Capital Stock(*Fixed*_*it*_) are also introduced as control variables to measure the spatial effects of the level of market integration and economic growth. Ultimately, spatial analysis models were utilized to perform two analyses: fixed effects and decomposition effects. A theoretical research framework on the spatial relationship between economic development and integrated market development is constructed, which provides some innovative research perspectives on the relationship between economic development and integrated market development from the perspective of the attributes of geospatial relationships. Simultaneously, as one of the engines of China’s economic development, the PRD city cluster is representative of the spatial evolution of the economic development and market integration of China’s city clusters, and the development-oriented paths derived on this basis are somewhat universal.

## 2. Literature review

### 2.1 Theoretical foundations of market integration

In recent years, market integration has gradually become the focus of scholars’ attention, and the essence of the construction of market integration is to give full play to the decisive role of the market in resource allocation, market integration is not only a prerequisite for the construction of regional economic integration, but also an important prerequisite for the realization of inter-regional coordinated development. Market integration is a dynamic process that enables the intraregional flow of resources of various commodities and factors through institutional innovations, thereby increasing the total factor productivity of the region. For the study of market integration, Young found that the degree of segmentation of China’s domestic market increased during the 1980s by measuring and comparing commodity prices across China’s domestic regions [1]. Poncet reached the same conclusion as Young based on the measurement of agricultural commodity prices in 28 provinces and cities in China [2]. With the continuous advancement of national reforms, studies have found that the index of domestic market segmentation has gradually decreased, and research has used the method consistent with Young to measure the degree of China’s domestic market integration and found that the degree of China’s domestic market integration is increasing [3]. For academic discussion on the extent of regional and global economic integration, it is important to understand the extent and limitations of integration to allay concerns [4]. Additionally, the escalating significance of domestic economic standards and their integration effects are elucidated in another research [5]. Further insights in another study explained the reasons why and the extent to which the negotiating perspective of a country is determined by its ability to penetrate global value chains, embrace tariff reforms, and face the trade balance consequences [6]. Furthermore, for literature focusing on the Greater Bay Area, noteworthy contributions include Richardson BJ.’s study [7] and the study by Shang CS and Shen W [8].

### 2.2 Measurements of market integration

Most measures of market integration have focused on the horizontal level, while a few scholars have measured market integration from a spatial perspective. To measure the level of market integration at the horizontal level, there is the trade flow approach, which examines the degree of market segmentation through trade flow data [9]. However, with the continuous development and deepening of research, Xu X. suggested that interregional trade flows are affected by some uncontrollable factors such as economies of scale, and factor endowment structure, and thus cause price changes [10]. Another is the relative price index method, in which prices measure the efficient allocation of market resources and act as a signal. In the process of economic development commodity price information can show the efficiency of production of commodities as well as trade flows in the market. The law of one price, modified on the basis of the "glacial" cost model, states that prices in two regions may end up being identical due to differences in transaction costs, but changes in the volatility of commodity prices between the two regions will still show a more pronounced correlation and that the interregional price differentials are a smooth stochastic process. The trend of regional market integration can be calculated using the change in relative price variance Var($\frac{P_{i}}{P_{j}}$)[11]. When the variance Var($\frac{P_{i}}{P_{j}}$) narrows over time, it indicates that the range of relative prices between markets is narrowing, the "glacial" cost c is decreasing, and the no-arbitrage band [$1-c,\frac{1}{\left(1-c\right)}$] is narrowing. The degree of market integration between the two regions can be considered to have increased, as trade barriers between the two regions gradually weakened and factors limiting market integration diminished.

### 2.3 Transmission path of market integration to economic growth

There are two main views of scholars who have studied the effect of market integration on economic growth. For one, market integration positively affects economic growth. The other is that the effect of market integration on economic growth is insignificant. First, for the view that market integration has a positive impact on economic growth, scholars have elaborated on the impact of market integration on economic growth in terms of resource allocation efficiency, knowledge and technology, talent and industrial agglomeration, innovation and development, and trade and investment. With the birth of the New Economic Geography, the endogenous interaction between financial externalities and agglomeration resulted from labor market sharing, market potential, and backward and forward linkages [12]. The New Economic Geography theory mainly explains the economic behavior of enterprises because of the Combined effect, as well as the inner mechanism and determinants of spatial agglomeration occurring in industries. According to the theory of Spatial Spillover, the economic development of a region has a spillover effect on its neighboring regions, and the closer the distance, the more obvious the spillover effect. As the level of regional market integration increases, interregional barriers will be gradually eliminated, thus promoting cross-regional factor flows and improving the efficiency of various resource allocations [13]. Henrekson. M & Torstensson. J developed an economic growth model and conducted a regression analysis of 115 countries and found that the process of EFTA and EC economic integration had a significant positive impact on their economic growth [14]. The free flow of production factors brought about by the increased level of market integration leads to the expansion of market demand and increased market competition. It facilitates the formation of economies of scale and the development of competition as well as learning, matching, and sharing mechanisms that lead to economic growth [15].

Second, consumers, producers, and researchers within a market have adequate channels of communication. The rapid transformation of demand into supply and the application of science to production will allow the diffusion of knowledge and technology across a large market, which will provide a sustained impetus for economic growth [16]. At the same time, market integration provides a realistic way for the diffusion of knowledge. Integrated markets provide the impetus for extensive coverage of communication facilities, while an easily accessible communication environment is necessary for innovation to arise [17]. The process of regional industrial agglomeration is accompanied by talent flow, thus further expanding the win-win effect of talent agglomeration and industrial agglomeration and laying the foundation for regional high-quality development. Due to the obvious externality of regional economic level and Combined effect, regions with a high degree of agglomeration and high economic quality can set a benchmark for the neighboring relatively backward regions through the Demonstration effect, and the neighboring regions can further promote the continuous improvement of regional factor correlation in the process of imitation, thus promoting the overall regional economic development [18–20]. Waltz found through a general dynamic equilibrium growth model that regional economic integration does have a catalytic effect on economic growth and that economic growth rates are related to several factors of regional economic integration, such as comparative production advantage and trade barriers, which can have either positive or negative effects on economic growth [21]. Dirk empirically examined whether and how regional market integration promotes convergence and economic growth among developing countries and found that regional market integration positively affects growth through increased trade and investment [22]. Opera and Stonica, Orlowski found that deep capital integration is necessary to support accelerated economic growth and that further market integration will provide access to capital financing, improve capital allocation, mitigate market and systemic risk, and promote real economic growth [18, 23]. Some scholars have argued that the effect of market integration on economic growth is insignificant. Andreas and Alexabder analyzed the East African market using satellite images measuring light emitted from the earth at night to measure economic activity and found that the economic growth effect was temporary and insignificant for the entire region [24]. Some scholars argue that China’s domestic market is falling into a state of market segmentation caused by local governments competing for benefits, resulting in slow market integration and stunted economic growth [1, 2]. Brown analyzed the regional Free Trade Agreement (FTA) implemented by the US-Japan using the CGE model and showed that the integration agreement had a positive but insignificant effect on the economic growth of both Japan and the US [25].

In summary, the existing research on the impact of market integration on economic growth has been relatively rich, but there are still the following shortcomings. On the one hand, in terms of research methods, spatial econometric models are less frequently used to analyze the spatial effects of market integration on economic growth. On the other hand, from the viewpoint of research objects, most of the literature on regional integration and economic growth effects has studied regional economic integration between countries, and less attention has been paid to economic integration within a country, especially the lack of research on the economic growth effects of market integration in a city cluster within a country. Finally, there are still relatively few studies on the insignificant effects of market integration on economic growth. Therefore, this paper explores the spatial effects of market integration on economic growth in the PRD city cluster, which will be beneficial to promote the high-quality economic development of the PRD city cluster.

## 3. Research methodology

### 3.1 Spatial econometrics methods

Macroeconomic variables among different cities in the PRD urban cluster may be spatially linked, and it is important to determine whether there is spatial autocorrelation among objects before conducting Spatial Econometric Modeling.

#### 3.1.1 Spatial autocorrelation analysis

Spatial autocorrelation is a prerequisite for spatial econometric analysis, and only after the existence of spatial autocorrelation is established can spatially correlated econometric analysis be performed on the data. Spatial autocorrelation refers to the potential interdependence between observations of some variables within the same distribution area, where spatial autocorrelation includes Global Spatial Autocorrelation and Local Spatial Autocorrelation. The global spatial autocorrelation is generally tested using the Moran Index test, which is calculated as follows.

$$I=\frac{\sum_{i=1}^{n}\sum_{j=1}^{n}W_{ij}(Y_{i}-\bar{Y})(Y_{j}-\bar{Y})}{S^{2}\sum_{i=1}^{n}\sum_{j=1}^{n}W_{ij}}$$ (1)

Where I denote the overall correlation between regions. $S^{2}\sum_{i=1}^{n}\sum_{j=1}^{n}W_{ij},\;\bar{Y\;}=\frac{1}{n}\sum_{i=1}^{n}Y_{i},\;Y_{i},Y_{j}$ denote the observations in the i-th and j-th regions, respectively. n represents the number of study areas, and W_ij_ is the spatial weight matrix. The interval of Moran’s index value is [-1,1], Moran’s I >0 indicates a positive spatial correlation, and the larger its value, the more obvious the spatial correlation. Moran’s I < 0 indicates a negative spatial correlation, and the smaller its value, the greater the spatial variation, and when Moran’s I = 0, the space is random.

The spatial weight matrix measures the degree of association between things, and there are two conventional settings, a simple binary adjacency matrix, and a distance-based binary spatial weight matrix.

The elements of the i-th row and j-th column of the simple binary adjacency matrix are

$$W_{ij}=\left\{\begin{array}{cc} 1, & When\;city\;i\;is\;adjacent\;to\;j\\ 0, & else \end{array}\right.$$ (2)

The elements of the i-th row and j-th column of the distance-based binary spatial weight matrix are

$$W_{ij}=\left\{\begin{array}{cc} 1, & the\;distance\;between\;cities\;i\;and\;j\;is\;less\;than\;d\\ 0, & else \end{array}\right.$$ (3)

There are two types of contiguity-based spatial weights in the concept of proximity: first-order proximity matrix and higher-order proximity matrix. The first-order proximity matrix assumes that spatial association occurs when two regions have a common boundary, that is when neighboring cities i and j have a common boundary denoted by 1, otherwise denoted by 0. There are two general methods of calculating Rook proximity and Queen proximity. The Rook neighborhood defines neighbors in terms of common boundaries only, while the Queen neighborhood includes neighbors with common vertices in addition to common boundary neighborhoods. It can be seen that the spatial matrix based on Queen proximity often has a closer correlation structure with the surrounding areas. Therefore, the spatial weight matrix based on Queen proximity is used for this paper. Local spatial autocorrelation, Moran scatter plot, and local correlation LISA plot are used in this paper to reveal the spatial internal structure and agglomeration characteristics of economic growth, and market integration.

#### 3.1.2 Spatial panel econometric model

**Fig 1:**
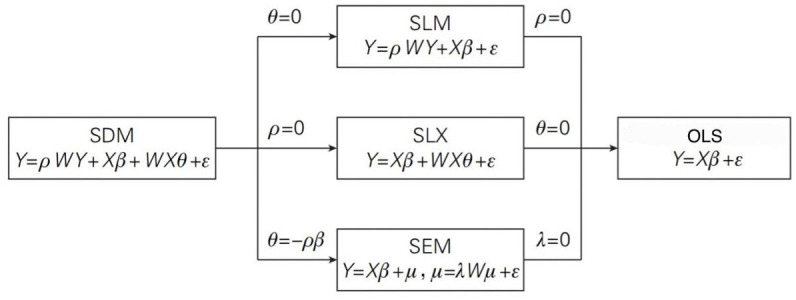
Relationship between SDM and SLM, SLX, SEM, and OLS.


For Spatial Econometric Models, Spatial Lag Models (SAR) and Spatial Error Models (SEM) are the two most commonly used models in spatial econometric studies. The decision of which model to choose depends on whether the dependent or independent variables have spatial interactions. SLM, SLX, and SEM models can be derived by imposing one or more conditional restrictions on the generalized nested spatial econometric model, and OLS models can be further derived. Fig 1 shows the specific process.

ϒ=ρWy+Xβ+θWX+μ (4)

The SDM model is:

ϒ=ρWϒ+Χβ+θWΧ+ε (5)

The SEM model is:

ϒ=Χβ+μ,μ=λWμ+ε (6)

#### 3.1.3 Selection of spatial econometric models

Because of the possible spatial correlation in the model, the Least Squares Parameter Estimation results may lead to the loss of validity and consistency of the estimated parameters, while the Great Likelihood Estimation can effectively solve this problem. According to the discriminant criterion proposed by Anselin: if neither LM-Error nor LM-Lag is significant in the test of spatial dependence then maintain the OLS model. If only the LM-Error is significant, select the SEM model, otherwise select the SLM model. If both are significant then a robust LM test is performed, if only Robust LM-Error is significant then the SEM model is chosen, otherwise, the SLM model is chosen [26]. When both are significant, referring to Lesage and Pace, both SEM and SLM models are applicable at this point, and the spatial panel econometric model with universal form can be chosen for analysis [17]. When the SDM model is selected, a simplified test of the SDM model using the LR test and the WALD test is required.

### 3.2 Analysis method of relative price index

This paper uses the relative price index method to calculate the PRD market integration index. The analysis method of the relative price index uses the test of relative price fluctuation range to measure the trend of market segmentation, which is a more effective use of prices as the most effective economic data and thus a true reflection of market integration. Samuelson’s iceberg transportation cost theory is the underlying theory of price law research to study market integration [27]. This theory is also a derivative of the law of one price. Its core idea is that the price ratio between any two markets for a commodity should be a stable value after taking into account objective factors such as transportation costs, and if this ratio exceeds a certain reasonable range, it represents a more serious trade barrier between these two markets. This concept can be more clearly stated by a simple mathematical expression. Suppose the price of a good in market A is *P*_*a*_, and the price at market B is *P*_*b*_. Suppose A is the production location of the product, then let *Y*_*b*_ be the location where the consumer is located, *Y*_*a*_ is the location of the production location, and *f* is the parameter of the interval (0, 1), which represents the unit transportation cost from market A to market B. At this time, the price of the commodity in market B is

$$P_{b}=P_{a}e^{\left(f\left|P_{b}-P_{a}\right|)\right.}$$ (7)

From the formula (7) can be seen, that any goods from one place to another place always have to incur certain costs, therefore the value of goods in the place of origin is always higher than the place of consumption, which does not indicate that the price of the place of consumption to be lower than the place of production, only that the goods in the process of transportation will have some losses. Take the example of a unit of goods arriving from A to B. Upon arrival at B, only 1-*f* unit of what would have been a unit remains. The smaller *f* is the smaller the transaction cost between the two places, which *f* does not simply refer to transportation costs, but is a collection of transaction costs. The iceberg transportation cost model suggests that the values of *P*_*b*_/*P*_*a*_ will have a corresponding range. This range represents the strength of the trade barriers between the two markets. If the trade barriers are small, the relative price takes a small value and the two markets are integrated and consolidated. On the contrary, a larger range of relative price-taking indicates that the two markets are fragmented. According to Parsley and Wei, the nine cities were first paired in pairs and differentiated the relative prices of each of the seven price indices mentioned below for each city [28]. Obtaining $\Delta Q_{ij}^{k}$ and then taking the absolute value of $\left|\Delta Q_{ijt}^{k}\right|$ for it, the final formula to be applied is as follows.


$$\Delta Q_{ijt}^{k}=\text{ln}\;\left(\frac{P_{it}^{k}}{P_{it-1}^{k}}\right)-\text{ln}\;\left(\frac{P_{jt}^{k}}{P_{it-1}^{k}}\right)$$ (8)

Further transforming the equation into:

$$\Delta Q_{ijt}^{k}=\text{ln}\;\left(\frac{P_{it}^{k}}{P_{jt}^{k}}\right)-\text{ln}\;\left(\frac{P_{it-1}^{k}}{P_{jt-1}^{k}}\right)$$ (9)

where *k* represents the category of the commodity, *i* and *j* are the respective cities, and *t* is the selected year. Since the price indices are selected from the statistical yearbook, it is convenient to use the above formula to exploit its chain characteristics. Also taking the logarithm alleviates the data heteroskedasticity and skewness well, and taking the absolute value also solves the placement order problem in city pairing. A total of 9 cities with 5 price indices for 10 years gives us 600 relative prices in differential form (10* 12*5), with 12 paired cities. Pairing each city with adjacent cities one by one. They are Foshan—Zhaoqing, Foshan—Zhongshan, Foshan—Jiangmen, Zhuhai—Zhongshan, Zhuhai—Jiangmen, Guangzhou—Huizhou, Guangzhou—Dongguan, Guangzhou—Foshan, Guangzhou—Zhongshan, Shenzhen—Dongguan, Huizhou—Dongguan, Huizhou—Shenzhen. Since we take the absolute value of the relative price for this differential form $\left|\Delta Q_{ijt}^{k}\right|$. Thus the solid heterogeneity associated with a particular good can be eliminated using the following equation.

$$\left|\Delta Q_{ijt}^{k}\right|-\left|\Delta \overset{¯}{Q_{t}^{k}}\right|=\left(a^{k}-{\bar{a}}^{k}\right)+\left(\varepsilon_{ijt}^{k}-{\bar{\varepsilon }}_{ijt}^{k}\right)$$ (10)

where *a*^*k*^ represents the systematic bias due to fixed effects and $\varepsilon_{ijt}^{k}$ represents the price differences caused by different environments with individual cities. Eq (10) is specified as given a specific year *t*, the commodity-specific price index *k* of $\left|\Delta Q_{ijt}^{k}\right|$. The differential relative price $\left|\Delta Q_{ijt}^{k}\right|$ obtained for each of the 12 pairs is then subtracted from $\left|\Delta \overset{¯}{Q_{t}^{k}}\right|$. Use $q_{ijt}^{k}$ to represent the right-hand side of Eq (10), and determine the range of price fluctuations in different city markets by calculating the variance of $q_{ijt}^{k}$. Further, the degree of regional market segmentation can be judged. When the variance representing the range of fluctuations is larger, that is, the larger Var(*q*_*ij*_) is, the more severe the market trading conditions are and the market segmentation is serious.

### 3.3 Absolute deviation method of the average wage of employees on duty

Assuming that the number of cities in the examined region is n, i represents the examined city, t is the examined period, and w is the actual wage level in the city. Then the average wage in the labor market of all towns in the region is $\bar{w}$. Provided further that the deviation of city j relative to the regional average labor market wage is *u*_*j*,*t*_. The absolute mean deviation, a measure of regional labor market integration examined, is *U*_*t*_. The arithmetic relationship between the above variables can be expressed as follows.


$$\bar{w}=\sum_{i=1}^{n}\;w_{i,t}/n$$ (11)

$$u_{j,t}=w_{j}-\sum_{i=1}^{n}\;w_{i,t}/n$$ (12)

$$U_{t}=\sum_{i=1}^{n}\;\left|u_{j,t}\right|/n$$ (13)

### 3.4 Coefficient of variation method

In dealing with the weights of each indicator, the coefficient of variation method is used considering the elimination of subjective factors in the determination of weights, and the specific calculation formula is as follows.

$$V_{j}=\frac{\sigma_{j}}{\overset{¯}{x_{j}}}(j=1,2,\cdots ,n)$$ (14)

$${\;\omega }_{j}=\frac{V_{j}}{\sum_{j=1}^{n}\;V_{j}}(j=1,2,\cdots ,n)$$ (15)

where $V_{j}$ is the coefficient of variation of the jth indicator, $\sigma_{j}$ is the standard deviation of the jth indicator, and $\overset{¯}{x_{j}}$ is the weight of the jth indicator. The market integration evaluation methodology is as follows.

$$U=\sum \omega_{j}x_{ij}^{'}$$ (16)

where *U* is the composite score, *ω*_*j*_ is the weight of the jth indicator, and $x_{ij}^{'}$ is the standardized indicator data.

## 4. Research design

### 4.1 Variable description

In this paper, we use economic growth as the explanatory variable, the aggregate market integration index as the core explanatory variable, and incorporate various control variables in the model by considering the degree of economic openness, human capital, consumption level, government spending scale, and social capital stock.

#### 4.1.1 Explained variable

Regarding the selection of variables for the level of economic growth, according to Luigi Guiso and the American economist Samuelson in Economics, economic growth is "the annual growth rate of a country’s real national output (GDP)" [29]. At the same time, considering that the GDP per capita index can better reflect the fair and balanced goals pursued by economic development, this paper adopts GDP per capita to represent the economic growth level index.

#### 4.1.2 Explanatory variable

The core explanatory variable in this paper is the Market Integration Total Index (*Intet*_*it*_). In this paper, market integration is divided into segmental market integration such as goods, labor, and capital.

1. **Commodity market integration**
  According to the connotation of the relative price index method, the commodity sector chosen should be able to encompass, to some extent, various aspects of the production and life of the people. Considering the availability of data, this paper selects the 2010–2019 Consumer Price Classification Index (CPI), which specifically includes the five major categories of food, tobacco, alcohol and supplies, clothing, medical and health care supplies, and transportation and communication to measure commodity market integration.
2. **Labor market integration**
  Labour market integration mainly refers to the formation of a unified labour market between regions under the mechanism of free movement of labour, to achieve a reasonable and optimal allocation of labour resources within the region. Borjas’s study shows that Mexican immigrants move across U.S. states more sensitively to wage differentials than U.S. natives, thus Mexican immigrants can accelerate inter-regional wage convergence and have a significant effect on inter-regional labor market integration [30]. Behrens and Sato analyzed the effects of wage structure, and GDP per capita on labor market integration and migrant skill formation and found that labor market integration is influenced by wage rates in the short run and indirectly due to migration in the long run [31]. This study uses the absolute deviation values of the average wages in each city over the years to measure the labor market integration in the PRD city clusters.
3. **Capital market integration**
  Martin Feldstein and Charles Horioka first modeled the FH test in testing the free flow of capital among 16 OECD countries and the degree of international financial market integration [32, 33]. The basic idea of the model is mainly based on the impact of capital flows on the correlation between regional savings and investment. Should there be barriers to the interregional flow of capital, local savings would be confined to financing investments within the region itself. Consequently, regional investments would become heavily dependent on these local savings, leading to a pronounced correlation between the two within that specific region. Conversely, when capital can freely move across regions, savings from any given region can be channeled towards areas offering higher returns. Similarly, regions with promising investment opportunities can benefit from savings originating elsewhere. Under such circumstances, the correlation between savings and investment within a specific region becomes negligible.

Drawing from the Harrod-Domar model, a cornerstone of classical economic growth theory attributed to Adam Smith, capital accumulation is underscored as a predominant factor influencing economic growth [34, 35]. Therefore, the balance of various deposits per capita of financial institutions at year-end and the balance of various loans per capita of financial institutions at year-end are selected to measure the capital market.

Meanwhile, according to (Samuelson, 1954), the relative price index method was used to measure the commodity market integration index and capital market integration index for nine cities in the PRD from 2010 to 2019. The absolute deviation method of the average wage of employed workers is used to calculate the labor market integration index. The coefficient of variation method is applied to determine the weights, and then the total market integration index is obtained.

#### 4.1.3 Control variables

This paper introduces Economic Openness(*Open*_*it*_), Human Capital(*Pers*_*it*_), Consumption Level(*Con*_*it*_), Scale of Government Spending(*Gov*_*it*_) and Social Capital Stock(*Fixed*_*it*_) as control variables. The description of each variable is shown in Table 1 below.

**Table 1:** Variable descriptions.

Variable Type	Variable Indicators	Measurements	Symbol
Explained variable	Economic Growth	GDP per capita	RGDP_it_
Explanatory variable	Market Integration	Market IntegrationTotal Index	lntet_it_
	Economic Openness	Imports and exports per capita	Openit
	Human Capital	Number of college students per 10,000 people	Pers_it_
Control variables	Consumption Level	Regional total retail sales of consumer goods as a proportion of GDP	Con_it_
	Scale of Government Spending	Local general public budget fiscal expenditure as a proportion of GDP	Gov_it_
	Social Capital Stock	Social fixed capital investment as a share of GDP	Fixed_it_

1. **Economic openness(*Open***_***it***_**)**
  Economic openness reflects the impact on economic growth of the extent of regional trade in imports, exports, and mutual investment. According to Paul R. Krugman, a prominent figure in the field of economic geography, international trade plays a pivotal role in facilitating technological spillovers that boost economic growth. Given the significant influence of international trade on regional economic expansion, this paper utilizes the per capita imports and exports metric as an indicator of economic openness [36].
2. **Human capital(*Pers***_***it***_**)**
  Differences in human capital are a prerequisite for regional division of labour and cooperation, and one of the factors leading to different levels of market integration between regions. The enhancement of human capital is conducive to narrowing regional differences and promoting the development of market integration. For the measurement of human capital, according to Barro RJ and Lee JW, this paper uses the average number of college students per 10,000 people to measure the level of human capital [37].
3. **Consumption level(*Con***_***it***_**)**
  Consumption plays a pivotal role in driving economic growth, and an uptick in consumption levels fosters the broadening of social production. This study employs the Regional total retail sales of consumer goods as a proportion of GDP as an indicator of consumption levels.
4. **Scale of government spending(*Gov***_***it***_**)**
  The scale of government spending reflects the degree of government regulation of local economic development, and according to macroeconomic theory and Wagner’s Law, government spending is an important initiative for economic growth. Therefore, this paper uses Local general public budget fiscal expenditure as a proportion of GDP to measure the size of government expenditure.
5. **Social capital stock(*Fixed***_***it***_**)**
  Social Capital Stock is conducive to promoting factor mobility and more efficient resource allocation, which in turn affects regional economic growth. Therefore, the indicator of social fixed capital investment as a share of GDP is used to measure the level of social capital investment.

### 4.2 Model construction

To investigate the spatial effect of the level of market integration on economic growth, the following model is constructed based on a spatial panel econometric model.

1. **Spatial durbin model**
  To study the spatial effects of the economic growth of neighboring cities in the PRD urban agglomeration and also to analyze the impact of the level of market integration of neighboring cities on the economic growth of the observed cities, the SDM model is first constructed.
$$ln{RGDP}_{it}=a_{0}+\rho Wln{RGDP}_{it}+{lntet}_{it}\beta +W{lntet}_{it}\theta +bK_{it}+\varepsilon_{it},\varepsilon_{it}\sim N(0,\sigma_{it}I)$$ (17)
  where RGDP_it_ is the level of GDP per capita in year t in the ith city, ρ is the coefficient of the spatial lag term WlnRGDP_it_, W is the spatial weight matrix, β is the coefficient of the explanatory variable, θ is the coefficient of the spatially lagged term of the explanatory variable, b is the coefficient of the control variable, K_it_ is the control variable, and ε_it_ is the random disturbance term. To mitigate heteroskedasticity, natural logarithms are taken for absolute values of variables RGDP_it_, *Open*_*it*_, and *Pers*_*it*_ in this paper.
2. **Spatial lag model**
  For each city in the PRD city cluster, the economic development of one city will be influenced by the level of economic growth of neighboring cities. Construct an SLM model suitable for this paper. When θ = 0 in Eq (4), the SLM model is obtained as follows.
$$ln{RGDP}_{it}=a_{0}+\rho Wln{RGDP}_{it}+{lntet}_{it}\beta +bK_{it}+\varepsilon_{it},\varepsilon_{it}\sim N(0,\sigma_{it}I)$$ (18)
3. **Spatial error model**
$$ln{RGDP}_{it}=a_{0}+{lntet}_{it}\beta +bK_{it}+\mu_{it},\mu_{it}=\lambda W\mu_{it},\varepsilon_{it}\sim N(0,\sigma_{it}I)$$ (19)
  Many factors that affect economic growth other than the degree of economic openness, human capital, and consumption level selected in this paper, such as the influence of the environment in which the city is located. To study the impact of market integration and unobservable factors on economic growth, the SEM model is constructed. In Eq (4), when θ = −ρβ, the SEM model equation can be obtained as follows.
  λ is the spatial error autoregressive coefficient, Wμ_it_ is the spatial lag term of the random error term, and ε_it_ is the random error term of the normal distribution.

### 4.3 Data sources and descriptive statistics

The data used in this paper are mainly from the Guangdong Provincial Statistical Yearbook for the calendar years 2010–2019, and the descriptive statistics of each variable are shown in Table 2 with the following sources: This study examines the market economy and the spatial progression of economic development across nine cities in Guangdong Province: Guangzhou, Shenzhen, Foshan, Huizhou, Dongguan, Zhuhai, Zhaoqing, Jiangmen, and Zhongshan. The data were selected from the above nine cities from 2010–2019 for GDP per capita, Market Integration Total Index, Imports and exports per capita, Number of college students per 10,000 people, Regional total retail sales of consumer goods as a proportion of GDP, Local general public budget fiscal expenditure as a proportion of GDP and Social fixed capital investment as a share of GDP as the foundation data of the study. The data were obtained from the statistical data of the Guangdong Provincial Statistical Yearbook (http://stats.gd.gov.cn/gdtjnj/), and the specific statistical characteristics of the variables are shown in Table 2 below. This paper intends to measure the development level of market integration and economic growth level of the PRD urban agglomeration, explore the spatial development structure and evolutionary impact mechanism of market integration and economic growth of the PRD urban agglomeration, and propose targeted paths to enhance the economic development level of the PRD urban agglomeration.

**Table 2:** Statistical characteristics of variables.

Variables	Sample	Average value	Standard deviation	Minimum value	Maximum value
lnRGDP	90	11.2465	0.4253	10.1216	11.9821
lntet	90	6.2816	0.1863	6.0694	6.6979
lnOpen	90	9.2797	0.8874	7.5896	11.4000
lnPers	90	4.9348	0.9363	3.4368	6.6837
Con	90	0.0273	0.0173	0.0052	0.0743
Gov	90	0.0085	0.0063	0.0020	0.0304
Fixed	90	0.0261	0.0111	0.0062	0.0567

## 5. Empirical analysis

### 5.1 Empirical study on the evolution mechanism of the spatial pattern of economic growth and market integration in the PRD region

#### 5.1.1 Global spatial autocorrelation analysis

Based on Eqs (1) and (3), the Moran index values of GDP per capita in the PRD urban agglomeration from 2010–2019 are measured, and the overall correlation of the PRD urban agglomeration region is studied. (The economic growth of the PRD urban agglomerations is significantly and spatially positively correlated globally.) From Table 3, the Moran index values of GDP per capita in the PRD urban agglomeration from 2010 to 2019 are all less than 0, and all pass the significance level test of 1% or 5%, indicating that the spatial distribution of GDP per capita in the PRD urban agglomeration has a significant negative spatial correlation, and the economic growth of each region in the PRD urban agglomeration is spatially clustered. A negative spatial correlation indicates that regions with higher economic levels are likely to have lower economic levels in their neighborhoods, and conversely, regions with lower economic levels have higher economic levels in their neighborhoods. Thus, the spatial distribution of GDP per capita in the PRD urban agglomeration is not random but shows a spatial convergence of regional GDP per capita. Using the same method to measure the Moran index values for the level of market integration in the PRD city cluster from 2010–2019, it is found that the spatial correlation is not significant, thus further analysis of the local correlation is needed.

**Table 3:** Moran index of GDP per capita in the PRD urban agglomeration, 2010–2019.

Year	Moran’s l	Z	GDP per capita (RGDP)	Year	Moran’s l	Z	P
			P				
2010	-0.487	-1.411	0.049	2015	-0.484	-1.422	0.053
2011	-0.487	-1.363	0.063	2016	-0.49	-1.452	0.047
2012	-0.478	-1.426	0.053	2017	-0.477	-1.41	0.047
2013	-0.463	-1.355	0.066	2018	-0.47	-1.388	0.05
2014	-0.467	-1.361	0.065	2019	-0.486	-1.423	0.051

#### 5.1.2 Local spatial autocorrelation analysis

**Fig 2:**
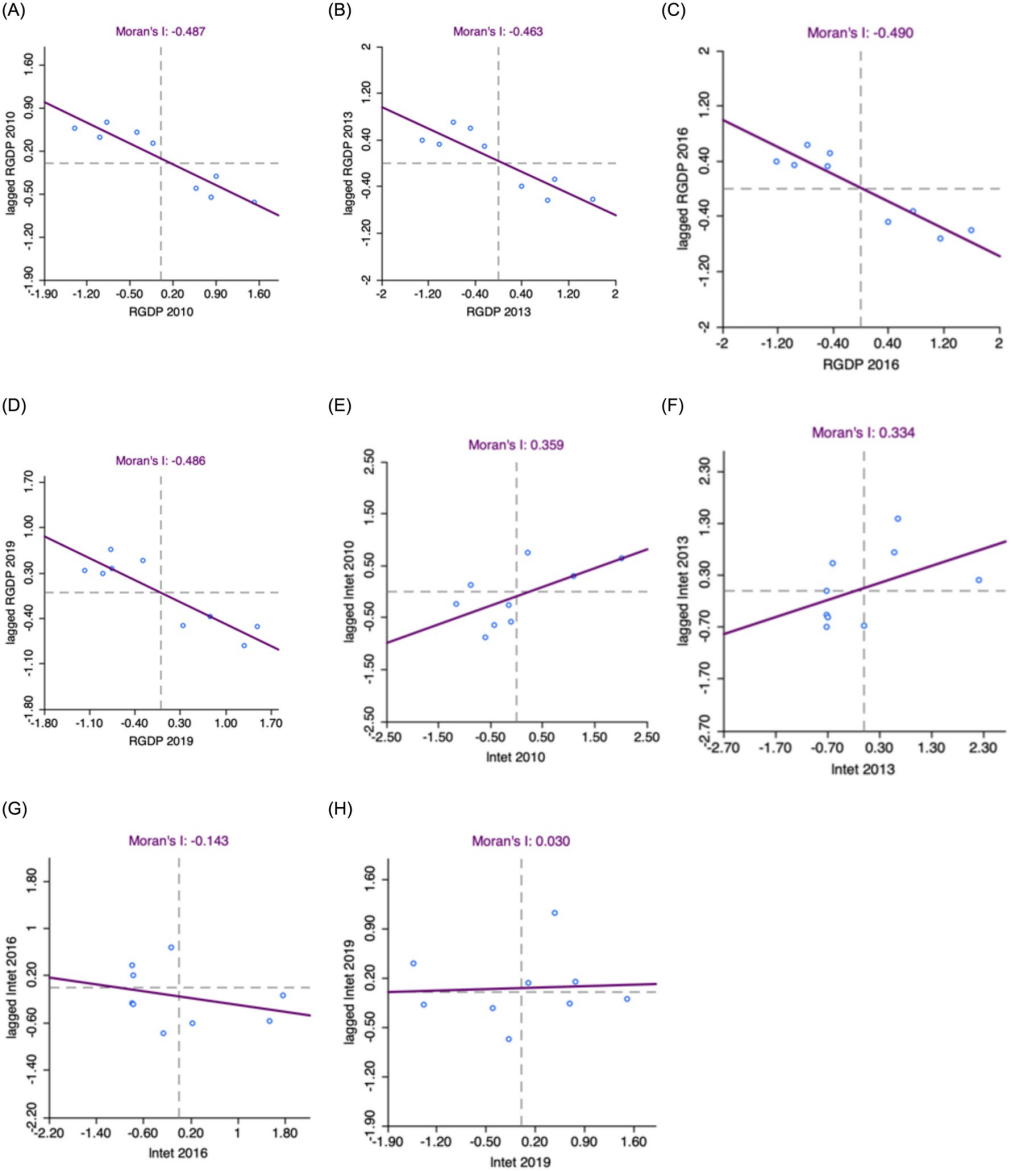
Scatterplot of the Moran Index of GDP per capita and market integration levels in the PRD city cluster, 2010, 2013,2016 and 2019. **A.** GDP per capita in 2010, B. GDP per capita in 2013, C. GDP per capita in 2016, D. GDP per capita in 2019, E. Market integration level in 2010, F. Market integration level in 2013, G. Market integration level in 2016, H. Market integration level in 2019.

**Fig 3:**
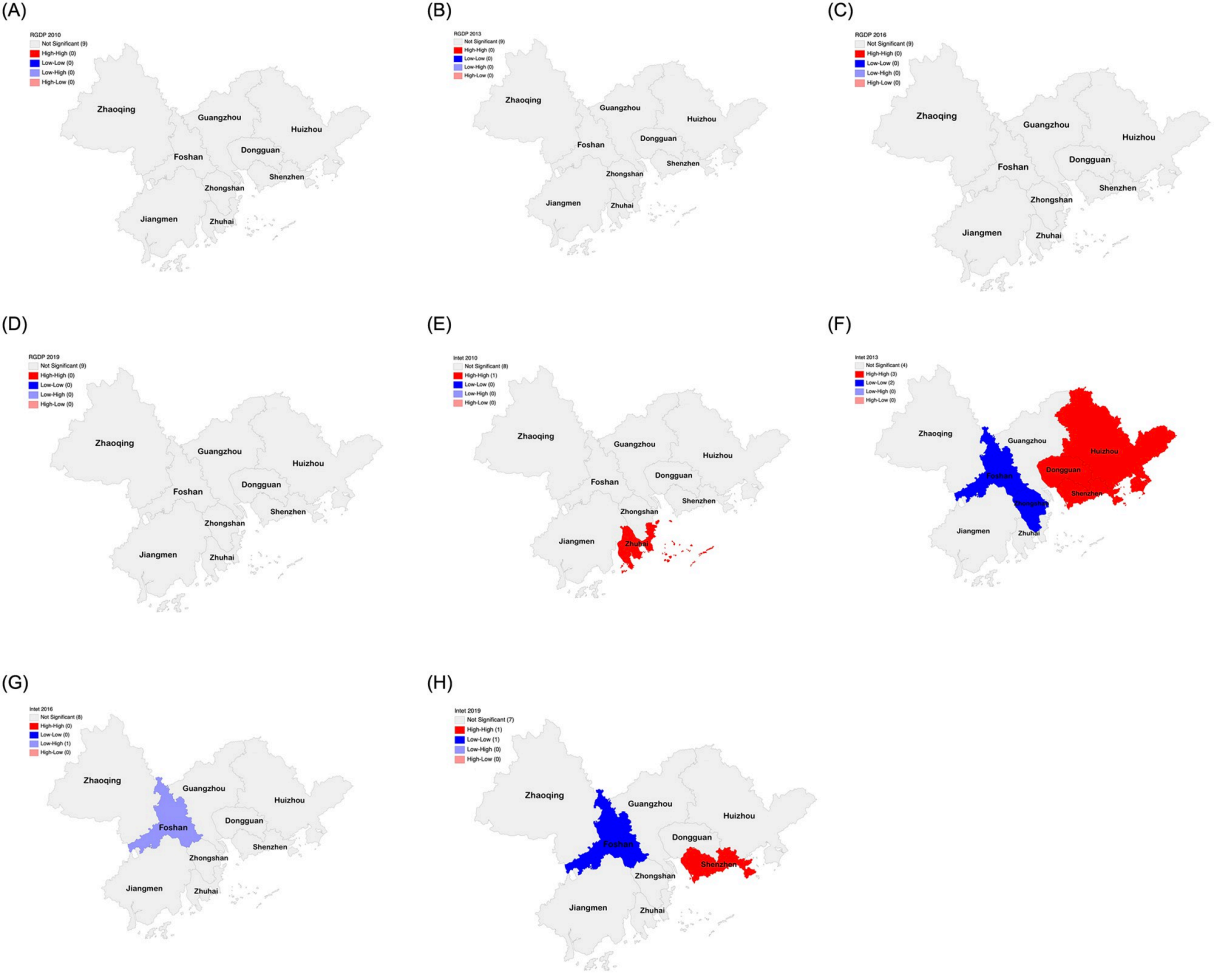
Local LISA agglomerations of GDP per capita and market integration levels in the PRD city cluster in 2010, 2013, 2016 and 2019 (p = 0.05). A. GDP per capita in 2010, B. GDP per capita in 2013, C. GDP per capita in 2016, D. GDP per capita in 2019, E. Market integration level in 2010, F. Market integration level in 2013, G. Market integration level in 2016, H. Market integration level in 2019.

To further understand the spatial agglomeration evolution characteristics of economic growth and market integration, scatter plots of the Moran index for the years 2010, 2013, 2016, and 2019 are presented in Fig 2. The scatter plot corresponding to the quadrant in which the city is located is also listed in Table 4. Additionally, to provide insight into the spatial concentration of GDP per capita and market integration for specific cities, the LISA plot with a 5% significance level is illustrated in Fig 3. In general, the local centers of economic growth in the PRD urban agglomeration show a spreading trend.

**Table 4:** Results of the multicollinearity test.

Variable	VIF	1/VIF
con	5.73	0.174464
fixed	4.91	0.203741
gov	3.89	0.256859
lnopen	2.39	0.419053
pers	1.15	0.866097
lntet	1.14	0.876012
Mean VIF	3.20	

From Fig 2A–2D, it can be seen that the GDP per capita of the urban agglomerations in the PRD in 2010, 2013, 2016, and 2019 is relatively concentrated in the second and fourth quadrants, which belong to the L-H agglomeration type and the H-L agglomeration type. The L-H agglomeration indicates that the cities surrounding the cities with lower levels of economic development are cities with higher levels of economic development, while the H-L agglomeration indicates that the cities surrounding the cities with higher levels of economic development are cities with lower levels of economic development. Also from Fig 2E and 2F, it can be seen that the spatial correlation index of the market integration level of the PRD city cluster in 2010 and 2013 is positive, and the market integration is relatively concentrated in the first and third quadrants, especially concentrated in the third quadrant, which belongs to the L-L agglomeration type, indicating that the city itself has a low level of economic development, and the surrounding cities also have a low level of economic development, reflecting the obvious trend of aggregation of high and low values of economic growth. As illustrated in Fig 2G, the market integration level for 2016 predominantly clusters within the second, third, and fourth quadrants, characterized as L-H, L-L, and H-L agglomeration types, respectively. Conversely, Fig 2H reveals that the 2019 market integration is largely concentrated within the first, third, and fourth quadrants, aligning with the H-H, L-L, and H-L agglomeration types, respectively.

As can be seen from Fig 3A–3D for the GDP per capita indicator, the spatial clustering of economic growth in each city in the PRD urban agglomeration is not significant in 2010, 2013, 2016 and 2019. It indicates that neither passed the local autocorrelation analysis test and the local spatial correlation is not significant.

From Fig 3E–3H, for the market integration level indicator, the market integration level of the PRD city cluster in 2010, 2013, 2016, and 2019 shows some localized agglomeration. Among them, Zhuhai entered the H-H agglomeration type in 2010 and was significant. In 2013, Huizhou, Dongguan, and Shenzhen displayed a significant H-H correlation, suggesting robust market integration within their metropolitan zones. Conversely, Foshan and Zhongshan manifested significant L-L correlations, pointing to a more subdued market integration within their respective metropolitan regions. In 2016, Foshan exhibited a significant L-H correlation. By 2019, this shifted to a pronounced L-L regional correlation in Foshan, denoting subdued market integration within its metropolitan confines. Concurrently, Shenzhen displayed a significant H-H regional correlation in 2019, signifying its accelerated development. This indicates an increased agglomeration among cities in the Shenzhen metropolitan zone and an enhancement in economic capacity. The H-H agglomeration type seen in Shenzhen in 2019 is predominantly observed in the coastal regions of the PRD, underscoring the potent influence and ripple effects of cities with advanced economic development on their neighboring counterparts.

### 5.2 Evolutionary mechanism of spatial effects of market integration on economic growth in the PRD urban agglomeration

#### 5.2.1 Rationale for the selection of spatial effects models

The Variance Inflation Factor (VIF) is the ratio of the variance in the presence of multicollinearity between the explanatory variables to the variance in the absence of multicollinearity. The larger the VIF, the more severe the covariance. The variance inflation factor of the independent variable x is denoted as VIF and it is calculated as:

$$VIF_{i}=\frac{1}{1-R_{i}^{2}}i=1,2,\cdots ,k$$ (20)

where $R_{i}^{2}$ is the decidable coefficient from a linear regression of k-1 on the other explanatory variables with the ith explanatory variable as the explanatory variable. The method is the same as the auxiliary regression model test, except that a new indicator is constructed using the decidable coefficient VIF_i_. The larger the variance inflation factor, indicating that the closer $R_{i}^{2}$ is to 1, the stronger the degree of covariance between the ith explanatory variable and the other explanatory variables. If VIF_i_≥10, it indicates the existence of multicollinearity between the independent variables.

1. **Variance inflation factor (VIF) test**
  Due to the large number of selected variables, the variance inflation factor method is used to test for multicollinearity. Given the extensive variables chosen, we employed the variance inflation factor (VIF) method to assess multicollinearity. Table 4 reveals that the VIF values for the variables in this study do not surpass 10, indicating an absence of multicollinearity among them.
2. **Basis for the selection of spatial measurement models**
  Table 5 shows that the LM statistic passes the significance test at the 5% level, indicating that both the SEM model and the SLM model are applicable, and the more general SDM model can be selected for analysis. Further tests using Wald and LR statistics show that SDM models do not degenerate into SEM or SLM models. According to the Hausman test results, the statistics pass the significance test at the level of 10%, indicating that the fixed effect model is selected. In this paper, the SDM model of fixed effects is used to analyze the spatial effects of market integration on economic growth in the PRD urban agglomeration.

**Table 5:** Test of spatial dependence test model of PRD urban agglomeration.

Inspection method	Quantity of statistics	p-value
LM	172.45	0.000 0***
Hausman	41.28	0.000 0***
LR	104.11	0.000 0***
Wald	24.13	0.000 0***

#### 5.2.2 Results of empirical study on the evolution of the spatial effects of market integration on economic growth

In this paper, the SDM model of fixed effects is divided into three SDM models: space-fixed, time-fixed, and space-time (dual) fixed for comparative analysis. Table 6 shows the regression results, from the perspective of R^2^ value, the R^2^ value of the SDM model with spatio-temporal double fixed effects is the largest, which is 0.0571, indicating that the model has the best degree of fit. From the sigma^2^ value, the sigma^2^ of SDM model with spatio-temporal dual fixed effect is the smallest, which indicates that the model is stable. From the Log-likelihood value, the SDM model with spatio-temporal dual fixed effect has the largest Log-likelihood value, indicating the best explanatory ability. Therefore, it is more appropriate to use the SDM model with spatial-temporal double fixed effects to study the spatial effect of market integration economic growth in the PRD urban agglomeration.

**Table 6:** Fixed effects estimation of the spatial durbin model.

Variable	Space fixed	Time fixed	space-time (dual) fixed
Main			
lntet	0.1930**	-198.6298	-48.2086*
	(0.09228)	(141.0456)	(28.501)
lnOpen	0.1277 ***	0.4070***	0.1682***
	(0.04771)	(0.0566)	(0.0393)
lnPers	0.0662**	-0.0456	0.0654***
	(0.03123)	(0.0681)	(0.0237)
Con	-5.4690***	11.8843**	-5.3064**
	(2.0240)	(4.8772)	(2.4790)
Gov	7.6524**	-17.2988	7.1821***
	(3.2166)	(12.1070)	(2.5181)
Fixed	7.7463	8.5913	4.0822**
	(1.9167)	8.8667)	(2.0254)
Wx	`		
lntet	-0.0292	-0.1383***	0.0172
	(0.0314)	(0.0396)	(0.0342)
lnOpen	0.05611**	-0.0089	0.0645**
	(0.0248)	(0.0423)	(0.2888)
lnPers	0.0118	-0.1991***	0.0123
	(0.0120)	(0.0468)	(0.01823)
Con	6.8563***	1.1082	4.6252***
	(1.0364)	(3.1338)	(1.1121)
Gov	6.1447***	-11.6858	3.6909*
	(1.9414)	(7.7764)	(2.0504)
Fixed	1.6235	0.9237	0.6419
	(1.2099)	(3.5818)	(1.1601)
sigma2	0.0071***	0.266***	0.0008***
R2	0.0197	0.0011	0.0571
Log-likehood	158.3155	12.0091	181.8485

1. **SDM Model analysis of fixed effects**
  According to the SDM model analysis results of the time-space fixed effect in Table 6, the estimated coefficient of market integration level is -48.2086, which is significant at the 10% level, indicating that market integration is not conducive to the economic growth of the region. However, the estimated coefficient of the lagged term of the market integration level (W lntet) is 0.0172 and insignificant, indicating that the market integration process in this region has no obvious effect on promoting the economic growth of the surrounding areas. The possible reasons are as follows: first, in the process of promoting economic development through market integration in the PRD urban agglomeration, most resources are generally concentrated in central cities such as Guangzhou and Shenzhen, while the rapid development of surrounding cities and central cities is in sharp contrast. Despite the rapid development of central cities, market integration will exert a radiating driving effect on surrounding cities, but it will also have a certain "siphon effect". For example, the strong attraction of various resource elements in central cities such as Guangzhou will exert a certain restricting effect on the development of surrounding cities. Secondly, there are still administrative barriers among different cities in the PRD urban agglomerations, which lead to coordination difficulties, factor barriers lead to poor circulation within the urban agglomerations, and public service barriers lead to large gaps in public resources between cities, which hinder the flow of factor resources such as goods, labor, and capital, and thus make it difficult to exert the spillover effect on the economic growth of surrounding cities. In conclusion, the effect of market integration on the economic growth of each city in the PRD urban agglomeration shows a significant impact on the region and little impact on the surrounding areas. The estimation results of each control variable are different. First, the degree of economic openness, human capital and the scale of government expenditure all pass the positive significance test of 1%, indicating that the degree of economic openness, human capital, and the scale of government expenditure effectively promote the economic development of the region. At the same time, the regression coefficient of the interaction term of economic openness W lnOpen passes the significance test at the level of 5%, and the regression coefficient of the interaction term of government expenditure scale W Gov passes the significance test at the level of 10%, indicating that the improvement of economic openness and government expenditure scale in this region will promote the economic development of surrounding areas. However, the regression coefficient of the interaction term W lnPers of human capital level fails the significance test, indicating that the spillover effect of human capital level on the economic growth of surrounding areas is not obvious. Second, the consumption level passes the negative significance test, indicating that the consumption level has a significant inhibitory effect on the economic development of the region. Among them, the consumption level has an inhibitory effect on economic growth. The possible reason is that the lower income level affects people’s consumption level, and the lower level of consumption further affects economic growth and income level. From the regression results of its interaction term with spatial weight, the regression coefficient of the interaction term of consumption level passes the significance test of 1%, indicating that the consumption level has a negative spatial spillover effect on the surrounding areas. Third, the estimated coefficient of social capital stock passed the significance test of 5%, indicating that the increase of social capital stock in the region will promote the economic development of the surrounding areas, but its interaction term coefficient is not significant, indicating that the spillover effect is not obvious.
2. **Effect decomposition of SDM model**
  Based on the SDM estimation results of temporal and spatial fixed effects, this paper decomposes the spatial effects of various factors that affect the economic growth of the PRD urban agglomeration. Table 7 shows that: first, the direct effect of market integration on economic growth is negative, the indirect effect is positive, and the total effect is negative and insignificant, indicating that the process of market integration has an inhibiting effect on regional economic growth and the improvement of market integration level in neighboring areas has an insignificant promoting effect on regional economic growth. Second, the direct effects of economic openness and human capital on economic growth are positive, although the direct effects on economic development are not significant, which can indicate that the degree of economic openness and the level of human capital can promote economic growth in the region but the effect is not significant. Meanwhile, the indirect effect of economic openness is positive and the indirect effect of human capital is negative, both of which are insignificant, suggesting that the increase in the level of economic openness of neighboring regions attracts the concentration of factors in the region and promotes the economic development of the region, while the increase in the level of human capital inhibits the economic development of the region, but neither effect is significant. The total effect of both is significant, where the degree of economic openness passes the test at a 1% significant level and the human capital passes the test at a 5% significant level, and in general, the increase in the degree of economic openness and the level of human capital can promote the economic growth of the region. Third, for consumption level and social fixed capital, the direct effect of consumption level has a depressive effect on economic growth, and the direct effect of social fixed capital on economic growth is positive but insignificant. The total effect of both is positive and insignificant, which suggests that both the increase in the level of consumption and the level of social fixed capital contribute to the economic growth of the region, but the effect is not very significant. Fourth, for the size of government spending, the direct and indirect effects are positive and insignificant, and the total effect is positive and passes the test at the 1% level of significance, which indicates that the size of government spending has a positive effect on economic growth.

**Table 7:** Decomposition of SDM spatial effects of temporal fixed effects of market integration on economic growth.

Variable	Direct effect	Indirect effect	Total effect
lntet	-51.7893 (346.8081)	24.2916 (338.1286)	-27.4977 (20.0889)
lnOpen	0.1714 (0.4084)	0.0155 (0.4024)	0.1870*** (0.0441)
lnPers	0.0761 (0.0878)	-0.0198 (0.0892)	0.0563** (0.0286)
Con	-8.7071 (88.9210)	12.5636 (87.2395)	3.8565 (2.5287)
Gov	7.8502 (62.6840)	1.7525 (61.5278)	9.6027***(3.6764)
Fixed	6.0210 (27.4197)	-2.5935 (26.8749)	3.4275(2.6507)

## 6. Conclusion and insights

### 6.1 Conclusions of the empirical study

The conclusions of this paper are based on panel data from nine cities in the PRD from 2010–2019, and the spatial econometric model is used to analyze the spatial effects of market-integrated economic growth, leading to the following main conclusions.

1. The economic growth within the PRD city cluster exhibits pronounced spatial autocorrelation. Economic expansion in various regions of the PRD city cluster is spatially aggregated, with observable spillover effects from areas of higher economic development to those with lower levels of economic growth.
2. The spatial correlation regarding the level of market integration within the PRD urban agglomerations is inconspicuous. From the regression outcomes of the Spatial Durbin Model, it’s evident that market integration within the PRD city cluster acts as a restraint on the region’s economic advancement. Furthermore, the market integration process within the area has a negligible impact on spurring economic growth in adjacent regions. In the effect decomposition model, the overarching impact of market integration was notably significant.
3. Findings from the empirical analysis using the fixed SDM model indicate that economic openness, human capital, and the scale of government expenditure all contribute positively to the region’s economic growth. Both the degree of economic openness and the scale of government spending display a pronounced positive spillover effect. Conversely, the consumption level poses a substantial constraining influence on the region’s economic progression. Meanwhile, the impact of social capital stock on the regional economy is minimal, with its spillover effect being relatively indistinct.
4. The empirical results from the SDM effect decomposition model reveal that both the degree of economic openness and human capital have a positive direct impact on economic growth, with their overall effects being significant. The direct influences of consumption level and fixed social capital are positive, yet their aggregate effects, although positive, are not notably significant. For the scale of government expenditure, both direct and indirect effects are positive but lack significance. However, when considered in totality, the effect is both positive and notably significant.

### 6.2 Policy insights

In light of the empirical study’s results, this paper offers the subsequent recommendations:

1. The PRD urban agglomeration should leverage key economic hubs, particularly the pivotal roles of Shenzhen and Guangzhou, to bolster the collective economic progression of the PRD cluster. There should be an expedited push towards unified economic integration and development within the PRD urban agglomeration. This involves harnessing the influential economic reach of Guangzhou and Shenzhen to benefit other cities within the PRD ensemble. Efforts should aim to diminish regional disparities, foster cohesive economic growth between central cities and their peripheries, amplify the integrated economic influence and momentum of the metropolitan sphere, enhance the overall standard of tourism-driven economic development in the PRD cluster, and ultimately, bridge regional variances.
2. Eliminate market trade barriers and curb local protection. The emergence of counter-market-oriented behavior by local governments can interfere with the economic operating mechanism and impede the free flow of products and factors between regions. Break through the restrictions of administrative divisions to create smooth circulation channels, on the one hand, based on the regional stage of development and differences in resource endowments, combined with comparative advantages, the construction of a reasonable market access system, and strict control of the negative list of management can help break the explicit and invisible barriers between regions and unblock the flow of goods in the process. Enhancing the openness of the market economy, raising the level of human capital and the scale of government expenditure to promote vigorous economic development, actively guiding the cross-city mobility of economic factors between different cities within the PRD city cluster, enhancing the aggregation of urban industrial clusters and industrial chains, and linking up key economic development zones dispersed in various cities.
3. To stimulate robust economic growth, there is a need to amplify the openness of the market economy, elevate human capital, and augment the scale of government spending. It’s crucial to guide the inter-city movement of economic components within the PRD city cluster, bolster the consolidation of urban industrial hubs and their respective chains, and seamlessly connect pivotal economic zones scattered across the different cities.
4. Leveraging the distinct advantages of cities within the PRD city cluster requires the establishment of robust cooperative mechanisms. A sustainable regional collaboration framework is essential to eliminate obstacles impeding the movement of goods and economic factors. Local governments need to reassess their inherent market advantages with a long-term perspective and align these with tailored industrial strategies to bolster specialization and facilitate industrial advancement. Through this cooperative framework, a cohesive market characterized by a logical division of labor can be developed, thus hastening the diffusion of knowledge. To effectively navigate the city clusters, enterprises should be encouraged and assisted in active market competition, while reinforcing collaborative operational mechanisms among them. It’s crucial to adeptly synchronize labor division and collaboration across economies and industries of distinct cities, thereby sidestepping uniform competition among varying urban economies.
